# Bending Strength of Wood Treated with Propolis Extract and Silicon Compounds

**DOI:** 10.3390/ma14040819

**Published:** 2021-02-09

**Authors:** Magdalena Woźniak, Przemysław Mania, Edward Roszyk, Izabela Ratajczak

**Affiliations:** 1 Department of Chemistry, Faculty of Forestry and Wood Technology, Poznań University of Life Sciences, 60625 Poznań, Poland; izabela.ratajczak@up.poznan.pl; 2 Department of Wood Science and Thermal Technics, Faculty of Forestry and Wood Technology, Poznań University of Life Sciences, 60627 Poznań, Poland; przemyslaw.mania@up.poznan.pl (P.M.); edward.roszyk@up.poznan.pl (E.R.)

**Keywords:** scots pine, propolis, silicon compounds, bending strength, modulus of elasticity, natural preservatives

## Abstract

The modification of wood and its treatment with various preservatives may affect its mechanical properties, hence the knowledge of the character changes in wood caused by impregnation is of great importance. Therefore, the aim of the research was to determine the effect of impregnation, with the propolis-silane preparation (EEP-MPTMOS/TEOS) consisting of the propolis extract (EEP) and silicon compounds: 3-(trimethoxysilyl)propyl methacrylate (MPTMOS) and tetraethoxysilane (TEOS), on the bending strength of treated wood. Moreover, in the study wood treated with components of the propolis-silane formulation was used, namely 70% ethanol, the propolis extract, and silanes (MPTMOS/TEOS). In order to determine whether the impregnation of wood affects its long-term bending, creep tests were performed depending on the humidity. The impregnation of wood with the propolis extract and the propolis-silane preparation (EEP-MPTMOS/TEOS) contributed to the increase in modulus of rapture and work to maximum load values compared to the untreated wood. In dry wood condition, the wood treated with EEP and EEP-MPTMOS/TEOS was characterized by lower modulus of elasticity values than the control samples. In turn, in wet wood condition, wood treated with the propolis-silane preparation showed an increase in the MOE value. Moreover, the impregnation of wood had an influence on the wood creep process under bending loads. The treated wood was characterized by higher relative creep compliance than the untreated wood. The exception was the wood impregnated with EEP-MPTMOS/TEOS, which showed comparable relative creep compliance to the control samples. The presented results indicate that wood treated with a bio-friendly preparation based on propolis and silicon compounds can be used in various application and also in variable humidity conditions.

## 1. Introduction

Wood is a natural and renewable material, which has found numerous indoor and outdoor applications. However, its application, especially in external conditions can be limited by a few disadvantages, including moisture sensitiveness, low resistance to UV radiation, low dimensional stability, and bio-deterioration caused by termites, fungi, and marine borers [1,2]. In order to overcome the weak points of wood material, modification, including thermal, chemical, and impregnation with various substances, is applied. The strict toxicological requirements in European Union and growing ecological awareness of consumers result in an increasing interest in research aimed at developing new, biocides-free agents for wood preservatives. Among ecological products with potential application in wood protection, there are both synthesized chemical compounds and natural substances. Silver nanoparticles, ionic liquids or silicon compounds belong to chemical compounds with a low environmental impact and are described in the literature as potential and effective wood preservatives [3,4,5,6,7,8,9].

Silicon compounds improved many wood properties, such as hydrophobicity, dimensional stability, and photostability [6,9,10]. Moreover, silicon compounds, especially those with amino groups, increased the resistance of the treated wood against decay fungi and molds [4,6,11,12]. According to literature date, natural substances that are harmless to humans and the natural environment, such as chitosan, essential oils, natural oils or propolis, could also be applied in ecological wood protection [13,14,15,16,17].

Propolis is a natural beehive product with numerous biological activities, including antifungal, antibacterial, antiviral, and antioxidant [18,19,20,21]. The material from various geographical origin contains among others phenolic compounds (including flavonoids, phenolic acid and their esters), minerals, fatty acids, amino acids, and terpenes [18,21,22,23]. Propolis, mainly because of the inhibitory effect on the growth of various strains of fungi has been investigated as a potential wood preservative, both alone and as a complex formulation [17,24,25]. The wood protected with the propolis extract showed resistance against *Trametes versicolor* and *Neolentinus lepideus*, compared to the unprotected wood [17]. In turn, the wood impregnated with the propolis-silane preparation, consisting of the propolis extract and silicon compounds (3-(trimethoxysilyl)propyl methacrylate and tetraethyl orthosilicate) limited the activity of *C. puteana*, even if the wood was subjected to leaching procedure [26].

Scots pine (*Pinus sylvestris* L.) is a species widespread in the forests throughout Europe, including Polish forests [27]. The sapwood of pine is easy to process, therefore it has become a widely used raw material, also in outdoor construction. Moreover, references properties according to EN 384 [28] of Scots pine are well-known. However, the modification of wood and its treatment with various preservatives may affect its mechanical properties, hence the knowledge of the character of changes in wood caused by impregnation is of great importance [29,30,31]. The pine wood treated with ionic liquids is characterized by decreased bending strength, modulus of elasticity, and compression strength parallel to grain compared to the untreated wood [30]. In turn, wood impregnated with resin -1,3-dimethylol-4,5-dihydroxyethyleneurea showed lower values of modulus of rapture, impact bending strength, and shear than unimpregnated wood samples [29]. Mechanical properties (modulus of rupture and work to maximum load in bending) of wood modified with sorbitol and citric acid decreased compared to the unmodified control samples [32]. In turn, treatment of wood with mixtures of acetic anhydrate and acetoxy-functional siloxane did not affect its mechanical properties, namely bending test and the modulus of elasticity [33]. In addition, solvents used in preparation of wood preservatives may also influence the mechanical properties of wood [34,35]. Therefore, the aim of the research was to determine the effect of impregnation with 70% ethanol, the propolis extract, silanes (MPTMOS/TEOS), and the propolis-silane preparation (EEP-MPTMOS/TEOS) on the bending strength of treated wood. In order to determine whether the impregnation of wood affects its long-term bending, creep tests were performed depending on the humidity. These tests were carried out at a constant low (hygroscopic equilibrium in the laboratory) and constant high (wet wood) moisture of the wood.

## 2. Materials and Methods

### 2.1. Wood Samples

The investigated specimens with dimensions of 10 (T) × 10 (R) × 150 (L) mm^3^ were prepared from Scots pine (*Pinus sylvestris* L.) sapwood originating from the Wielkopolska Province, Poland. The samples were without growth inhomogeneity and showed no biotic infections. The average density of wood measured at 8% moisture content was 639 kg/m^3^, determined according to ISO 13061-2 [36]. In accordance with ISO 13061-1 [37], using a gravimetric method, the wood moisture content (MC) was determined.

### 2.2. Wood Treatment Preparations

For wood impregnation, 70% ethanol, propolis extract (EEP), silicon compounds, and propolis-silane preparation were used. The ethanolic extract of Polish propolis at 15% concentration (PROP-MAD, Poznań, Poland) was prepared in 70% ethanol. The silane preparation (MPTMOS/TEOS) consisting of 3-(trimethoxysilyl)propyl methacrylate (MPTMOS) and tetraethoxysilane (TEOS) at 5% concentration was prepared in 70% ethanol. The silicon compounds were purchased from Sigma Aldrich (Darmstadt, Germany). The propolis-silane preparation (EEP-MPTMOS/TEOS) contained 15% EEP, 3-(trimethoxysilyl)propyl methacrylate (MPTMOS), and tetraethoxysilane (TEOS) at 5% concentration.

### 2.3. Wood Treatment

The pine wood specimens were treated with 70% ethanol, propolis extract (EEP), silane preparation (MPTMOS/TEOS), and propolis-silane preparation (EEP-MPTMOS/TEOS) using the vacuum method. The samples were impregnated with tested solutions for 15 min under a vacuum condition of 0.8 kPa, followed by soaking for 2 h under atmospheric pressure. After treatment, wood samples were weighted to determine the solution’s uptake.

After impregnation, all the wood samples were conditioned to constant weight in room conditions (RH = 52 ± 2%; T = 20 ± 2 °C) for four weeks.

### 2.4. Static Bending Strength

The tests were performed on a universal mechanical strength testing machine ZWICK ZO50TH (Zwick/Roell, Ulm, Germany). Determination of mechanical parameters was carried out in a static bending test on samples with dimensions of 10 (T) × 10 (R) × 150 (L) mm^3^. The test machine output was used to calculate the modulus of elasticity (MOE) and work to maximum load (WML), as well as modulus of rupture (MOR). Static bending tests were conducted according to PN-77/D-04103 [38] for modulus of rupture (MOR) and PN-63/D-04117 [39] for modulus of elasticity (MOE), respectively. The distance between the supports during the test was 120 mm, while the load was placed exactly in the middle between the supports, on the radial surfaces. The rate of loading was chosen in a way to complete the test in about 90 s. In the test, ten wood samples of each variant treatment were used.

Modulus of rupture (MOR) was calculated as follows:

(1) $$\mathrm{MOR}=\frac{3F_{\max}L}{2{\mathrm{bh}}^{2}}\;(\mathrm{MPa})$$

where:

F_max_—maximum (breaking) force (N),

L—the distance between supporting span (mm),

b, h—width and height of the test samples (mm).

Modulus of elasticity (MOE) was calculated according to the equation:

(2) $$\mathrm{MOE}=\frac{3\left(F_{n+1}-F_{n}\right)L^{3}}{64{\mathrm{bh}}^{3}(f_{n+1}-f_{n)}}\;(\mathrm{MPa})$$

where:

F_n + 1_ − F_n_—the increment in load within the linear region of the load-deflection curve (N),

f_n + 1_ − f_n_—the increment in deflection (corresponding to F_n + 1_ − Fn), (mm).

The work to maximum load (WML) defines the amount of energy absorbed by the sample until it is destroyed. Its value is equal to the area under the curve σ-ε, and is expressed by the equation:

(3) $$\mathrm{WML}=\int_{0}^{\varepsilon }\sigma_{\max}{d\varepsilon }_{\max}\;(J)$$

where:

σ_max_—maximum stress; MOR (MPa),

ε_max_—maximum strain (mm).

After measurement of the bending strength, wood moisture content (MC) was also determined by gravimetric method according to the ISO 13061-1 [37] standard.

### 2.5. Rheological Wood Properties

**Figure 1:**
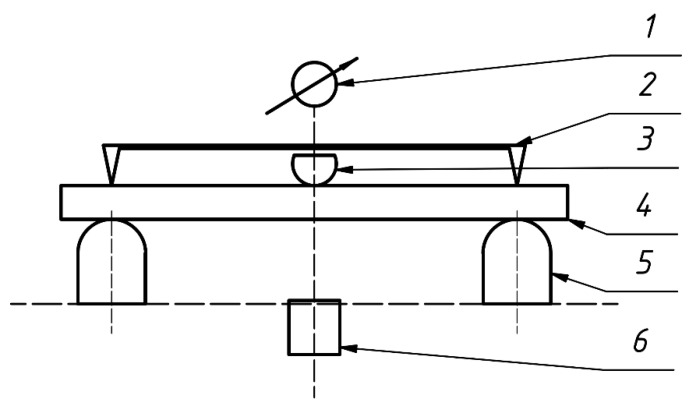
Scheme of prototype creep testing machine (1—micrometer; 2—deflectometr; 3—thrust; 4—sample; 5—suports; 6—load).

In order to demonstrate whether the impregnation of wood with the propolis-silane preparation and its components (70% ethanol, propolis extract and silicon compounds) shows an effect on its long-term bending, creep tests were performed depending on the moisture content. These studies were carried out at constant low (equilibrium moisture content in the laboratory) and a constant high (wet wood) wood MC. The wood creep process was investigated with the use of a prototype creep testing machine (Figure 1). The constant stresses, causing the bending of the wood, were caused by the suspension through the coupling system of the appropriate load, constituting 20% of its strength (MOR). The sample was placed on the stand of the prototype creep testing machine so that the thrust was exactly in the middle of its length, and the bending force was applied along the tangent to the annual rings. The distance between supports during the experiment was 120 mm. The deflectometer was set on the sample in such a way that the blades rested on the sample at the support points, and the measuring pin of the micrometer rests on the flat thrust surface. In the first stage of creeping, the micrometer indications were read every 15 s for the first 10 min, and then every half a minute. The research was conducted until 10 consecutive readings of deflection from the deflectometer were the same. In the test, three wood samples of each variant treatment were used.

### 2.6. Attenuated Total Reflectance Fourier Transform Infrared Spectroscopy (ATR-FTIR)

The spectra of wood, including control and treated samples were recorded by a Nicolet iS5 spectrophotometer (Thermo Fisher Scientific, Waltham, MA, USA) with Fourier transform and deuterium triglycinesulfate detector. The spectra were recorded at the 4000–400 cm^−1^, at a resolution of 4 cm^−1^, registering 32 scans.

### 2.7. Statistical Analysis

The obtained results were analyzed by the Dell^TM^ Statistica^TM^ 13.3 software (TIBCO Software Inc., Palo Alto, CA, USA). In order to determine the significant differences (*p* ≤ 0.05) in the investigated mechanical parameters of wood treated with different impregnating formulations, the analysis of variance (ANOVA) was used, followed by Turkeys’ honest significant difference (HSD) test.

## 3. Results and Discussion

In Table 1 the values of moisture content of wood, density before and after treatment, and retention of treated wood were presented.

The results of moisture content of control and treated wood after conditioning at RH = 52 ± 2%; T = 20 ± 2 °C showed that wood impregnation influenced this parameter. The wood treated with the propolis extract, silicon compounds, and propolis-silane preparation was characterized by around 2% decrease in the equilibrium moisture content compared to the control and EtOH-treated wood samples.

The average values of wood bending strength (MOR), modulus of elasticity (MOE), and work to maximum load (WML) are presented in Table 2.

The Table 2 shows the results for the wood in the hygroscopic equilibrium state in the laboratory (low moisture content) and above the fiber saturation point (wet state). When analyzing the MOR values, it can be observed that the impregnation of wood with the propolis extract (EEP) and the propolis-silane preparation (EEP-MPTMOS/TEOS) contributed to the increase in this value. This increase was statistically significant and averaged approximately 11%. In the case of wood impregnation with 70% ethanol and organosilicon compounds, the obtained differences were statistically insignificant compared to the control wood. This dependence occurs both in dry and wet wood (above the fiber saturation point), which may indicate the formation of permanent bonds between the components of propolis extract, organosilicon compounds, and wood. In wet wood, the difference between the control wood and the wood treated with EEP and EEP-MPTMOS/TEOS is even greater, because for EEP it is 23% but for EEP-MPTMOS/TEOS it is 75%. Both cases prove the positive effect of propolis preparations on the tested wood. The impregnation of wood with these preparations contributed to the reduction of wood hygroscopicity, which translates directly into an increase in the strength of such wood in a wet state. In the case of control wood, treated with 70% EtOH and MPTMOS/TEOS, the increase in moisture content above FSP contributed to a decrease in MOR by 53%, 59%, and 55%, respectively. The decrease in bending strength for dry and wet wood impregnated with the propolis extract was 48% and only 24% for the propolis-silane preparation.

Statistically significant differences can also be observed when analyzing the average MOE values. For this parameter, the treatment of wood with propolis preparations also contributed to a change in the mean values. In dry wood, pine wood modulus of elasticity values decreased. For wood protected with the EEP-MPTMOS/TEOS, the decrease in MOE was approximately 7.4% and it was the lowest value. Treatment of wood with all the tested solutions contributed to the reduction of wood stiffness. In wet wood, the effect of impregnation on the reduction of wood hygroscopicity can be observed. The average MOE values of impregnated wood do not differ significantly from the values for the control wood; the exception is wood after impregnation with EEP-MPTMOS/TEOS preparation. In this case, the modulus of elasticity increased by as much as 18%.

The highest values of work to maximum load (WML), i.e., energy of destruction, were characteristic for wood impregnated with propolis preparations (EEP and EEP-MPTMOS/TEOS). The average WML value for this type of protecting agents was on average 16% higher than in the control wood. For wet wood, the WML value of wood impregnated with EEP-MPTMOS/TEOS was over 56% higher than for the control wood sample. That much more energy will accumulate wood against destruction under bending loads.

Changes in mechanical parameters after wood treatment with various chemicals have already been described. However, the research concerned other agents than these described in this paper, e.g., ionic liquids [30], chitosan solutions [40], or resins [41]. The increase in MOR and MOE by 12% and 16%, respectively, was indicated by Ming-Li et al. [42], after impregnating the wood with sodium silicate solution compared to the control sample. A similar increase in the discussed mechanical parameters after wood impregnation by rosin compared to untreated wood was obtained by Dong et al. [43] and it was respectively 13% and 19%. Wood impregnation may also involve a reduction in mechanical parameters. Simsek et al. [44] contributed to the reduction of MOR by protecting wood with environmentally friendly borates. The wood treated with ionic liquids was characterized by lower values of bending strength and modulus of elasticity than control wood samples [30]. In turn, the wood impregnated with tetraethoxysilane (TEOS) showed comparable values of mechanical parameters (including MOR, MOE, WML, or impact bending) compared to the untreated wood [29].

**Figure 2:**
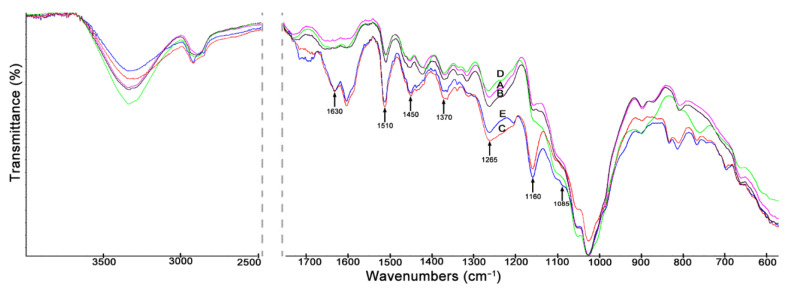
The ATR-FTIR spectra of Scots pine wood (A), wood treated with 70% EtOH (B), wood treated with EEP (C), wood treated with MPTMOS-TEOS (D), and wood treated with EEP-MPTMOS/TEOS (E).

The described changes in mechanical parameters of treated wood (especially in case of wood treated with the propolis extract and the propolis-silane preparation) could be due to various factors. Increase of MOR and WML values observed for wood treated with EEP and EEP-MPTMOS/TEOS could be connected with the formation of permanent bonds between wood and propolis preparation constituents. The changes in wood structure after impregnation with tested solutions were determined by ATR-FTIR and are presented in Figure 2.

The spectra of wood treated with EEP and EEP-MPTMOS/TEOS are characterized by the most significant changes. In the spectra of EEP- and EEP-MPTMOS/TEOS-treated wood, the band of O–H stretching vibration observed at 3454 cm^−1^ was narrower than in in the spectrum of control wood. This could be connected with the formation of hydrogen bonds between the propolis-silane preparation constituents and hydroxyl groups present in wood. Moreover, in the spectra of wood impregnated with EEP and EEP-MPTMOS/TEOS there are observed bands at 1630 cm^−1^ associated with C=C, C=O, and N–H vibrations originating from propolis constituents, mainly phenolic compounds and amino acids, and band at 1510 cm^−1^ indicating vibrations of skeletal C=C aromatic rings of flavonoids. The bands at 1450 and 1370 cm^−1^ connecting with bending vibration of C–H bond of flavonoids are presented in the spectra of propolis-treated wood samples. In addition, in the spectra of wood impregnated with EEP and EEP-MPTMOS/TEOS, the bands at 1265, 1160, and 1085 cm^−1^, assigned to vibrations of C–O, C–OH bonds of lipids, flavonoids or secondary and tertiary alcohols, that belong to the propolis constituents, are presented. Although, the bands at 1265 and 1085 cm^−1^ observed in the spectrum of wood treated with EEP-MPTMOS/TEOS could be also connected with vibration of Si–C and Si–O bonds from silicon compounds. In the case of the spectrum of wood impregnated with EEP-MPTMOS/TEOS, an overlap of the bands originating from the components of propolis and silanes is observed. The observed changes in the spectra of EEP- and EEP-MPTMOS/TEOS-treated wood indicate that constituents of EEP and EEP-MPTMOS/TEOS formed permanent bonds with the wood components. Moreover, in the spectrum of wood impregnated with MPTMOS/TEOS, the bands at 1265 and 1085 cm^−1^ are observed, which could indicate that silanes also form bonds with the wood, but the changes are less visible than in the spectrum of wood impregnated with the propolis-silane preparation. Therefore, we think that the silicon compounds used without propolis extract do not form permanent chemical bands with wood, but only fill the cell lumen or deposit on the wood surface. 

The increase in the bending strength observed at the wood moisture content equivalent to the equilibrium moisture content under standard conditions may be a result of lower equilibrium moisture content of the wood treated with EEP and EEP-MPTMOS/TEOS (Table 1). The impregnation of wood with the propolis extract and the propolis-silane preparation caused the reduction of wood hygroscopicity, which influence an increase in the strength of wood in a wet condition. With the increase in moisture content, the values of wood bending strength decrease in the entire hygroscopic range, and this decrease may amount to about 50% [45]. The increase of wood hydrophobicity caused by treatment with propolis extract, silicon compounds, and propolis-silane formulations was described in literature [11,46,47].

**Figure 3:**
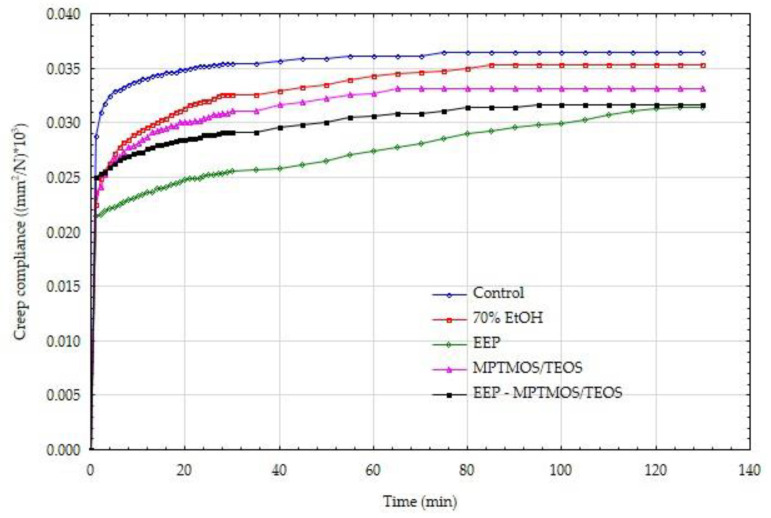
Creep compliance of dry wood, protected by various means.

**Figure 4:**
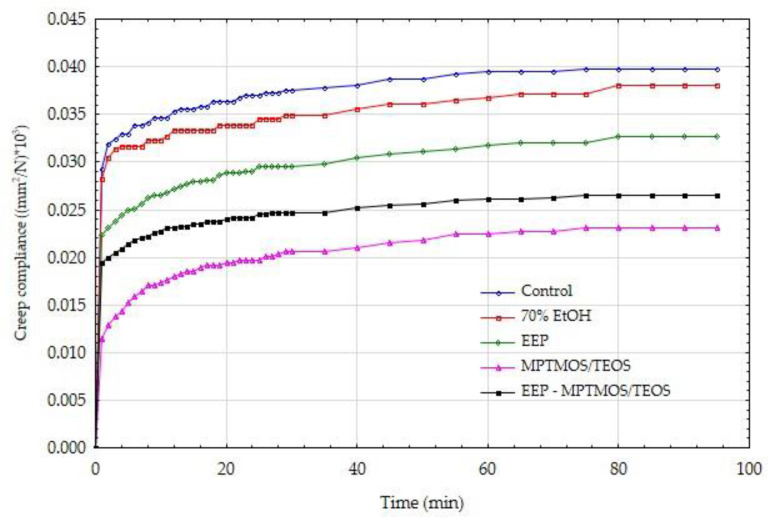
Creep compliance of wet wood, protected by various means.

In order to confirm the positive effect of the impregnating agents on the mechanical parameters, also in the long term, the wood creep process was carried out under bending loads. Figure 3 and Figure 4 show the creep tendency curves of wood. The values of creep susceptibility were calculated as the ratio of the average deformations recorded at a given moment in the process duration to the average value of the acting stress. The data presented in this figure show that the control samples with constant low and high moisture showed the highest susceptibility to creep.

Figure 3 and Figure 4 show that the obtained values of creep compliance for dry and wet wood condition are similar. However, it should be remembered that in the case of wet wood, much lower absolute load values were used. The assumed stress level was equal to 20% of the sample-breaking stress, i.e., it referred to the MOR for a specific sample protected with a different impregnating preparation. The creep compliance results are also burdened with some imperfections resulting from the immediate recording of the deflection arrow (at the moment of triggering the bending moment). For this reason, these results are presented further in the form of relative creep compliance.

In order to reduce the obtained results of creep compliance with the value of immediate compliance (for t = 0), the values of the so-called relative creep compliance, understood as the quotient of the creep compliance at a given moment in the process J (t) and the immediate compliance J (0):

(4) $$J^{\prime }\left(t\right)=\frac{J\left(t\right)}{J\left(0\right)}$$

**Figure 5:**
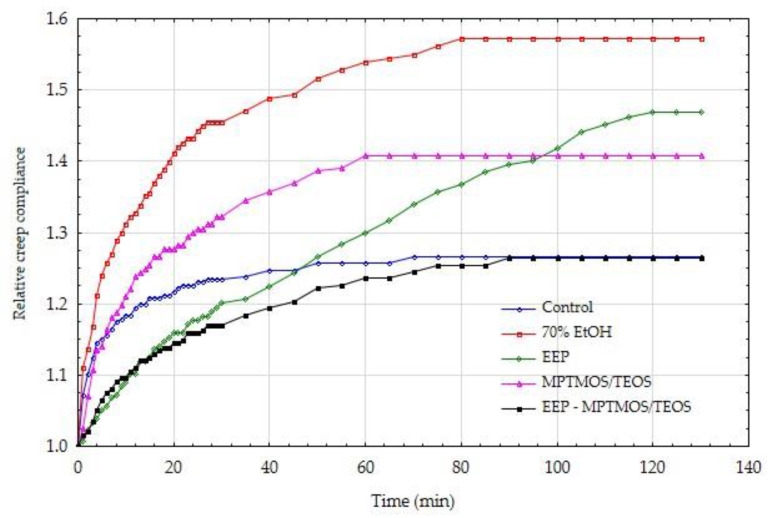
Relative creep compliance of dry wood, protected by various means.

**Figure 6:**
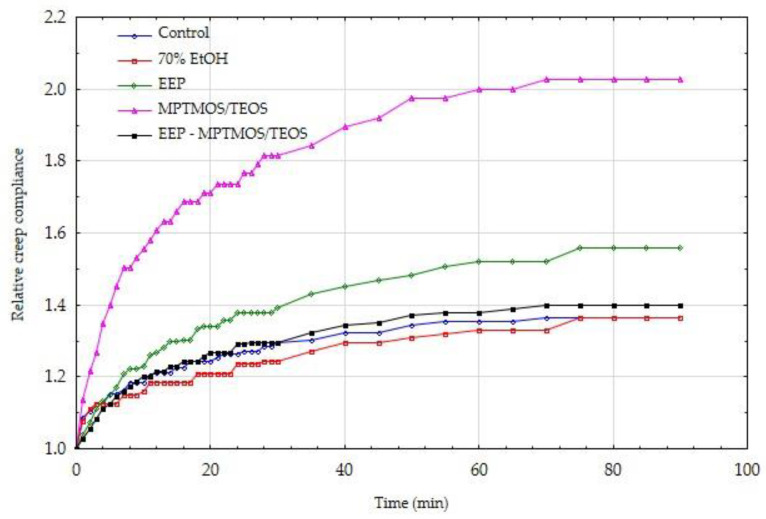
Relative creep compliance of wet wood, protected by various means.

The thus calculated results of the relative creep compliance for individual variants of the discussed experiments are presented in Figure 5 and Figure 6.

The analysis of the data presented in Figure 5 and Figure 6 shows that in the initial stage of creep, the values of relative creep compliance increased relatively quickly for all variants of the experiments. In dry wood, a large dispersion of individual variants can be observed. The control wood and wood protected with EEP-MPTMOS/TEOS solution showed the lowest relative creep compliance. The course of both curves is similar after approximately 60 min, but initially the control wood showed a higher relative creep susceptibility. The relative creep compliance curves in wood with a moisture content above the fiber saturation point are more closely related. The exception is the relationship for wood protected with the MPTMOS/TEOS solution, where the average value of the relative creep susceptibility is almost twice as high as the others.

Wood is a natural composite with a hierarchical structure. At the molecular level, cellulose, lignin, and hemicellulose are the main components of wood. Cellulose, which is an elastic polymer has a very small influence on the viscoelastic matrix, but lignin and hemicellulose present much more viscous behavior [48,49,50,51]. Moreover, lignin is the main component of middle lamella, which is a bonded layer connecting two adjacent cell wall and encrusting the cellulose skeleton [52]. The literature date indicate that aqueous ethanol solution dissolves a main part of lignin from the middle lamella, releasing the individual cells at sectioning [34,35]. The ethanol solution also caused the weakening of bonds between compound middle lamella and S1 layer [34,53]. The presented results indicate that wood samples impregnated with 70% EtOH showed the highest relative creep compliance in dry wood condition. Therefore, our hypothesis is that the ethanol–water mixture, which was used in the study as a solvent in impregnating formulations, could be one of the determinants of the changes occurring in the tests of treated wood creep. In turn, the lowest relative creep compliance observed in case of wood treated with the EEP-MPTMOS/TEOS could be connected with different mechanism. The constituents of propolis extract, especially when it is mixed with silicon compounds form bonds with wood, which is confirmed in the ATR-FTIR analysis. In this case, the negative effect of 70% ethanol could be compensated by permanent bonding of propolis constituents with wood components.

## 4. Conclusions

The paper presents the results of the effect of wood treated with the propolis-silane preparation (EEP-MPTMOS/TEOS) and its components (70% EtOH, propolis extract and silicon compounds) on bending strength of treated wood. The impregnation of wood with the propolis extract (EEP) and the propolis-silane preparation (EEP-MPTMOS/TEOS) contributed to the increase in modulus of rapture values, compared to untreated wood and wood treated with 70% EtOH and silicon compounds. This dependence was observed both in dry and wet wood conditions. In dry wood condition, the wood treated with EEP and EEP-MPTMOS/TEOS is characterized by lower modulus of elasticity values than control samples. In turn, in wet wood condition, wood treated with the propolis-silane preparation showed an increase in the MOE value. The wood treated with the propolis extract and the propolis-silane preparation in dry wood condition exhibited also an increase in the values of work to maximum load (WML). In turn, in wet wood condition, the WML value of wood impregnated with EEP-MPTMOS/TEOS was higher than the values for other wood samples. Moreover, the impregnation of wood had influence on wood creep process under bending loads. The treated wood was characterized by higher relative creep compliance than the untreated wood. The exception was the wood impregnated with EEP-MPTMOS/TEOS, which showed comparable relative creep compliance to control samples.

The described changes in mechanical parameters of treated wood (especially in case of wood treated with the propolis extract and the propolis-silane preparation) could be due to various factors. These changes may be connected with the formation of permanent bonds between wood and the constituents of propolis preparation, which was confirmed by ATR-FTIR analysis. The increase in the bending strength observed at the wood moisture content equivalent to the equilibrium moisture content under standard conditions may be a result of lower equilibrium moisture content of the wood treated with EEP and EEP-MPTMOS/TEOS. The impregnation of wood with the propolis extract and the propolis-silane preparation caused the reduction of wood hygroscopicity, which causes an increase in the strength of wood in wet condition. Also higher density of EEP- and EEP-MPTMOS/TEOS-treated wood can result in higher strength properties. In turn, the 70% ethanol used as a solvent in preparations, according to literature data dissolves a main part of lignin in the middle lamella, releasing the individual cells at sectioning, which could have an influence on the creep properties of the wood. Therefore, in case of wood treated with the propolis extract and the propolis-silane preparation the decrease of equilibrium moisture content and increase of density results in increased strength properties compared to untreated wood seems to be compensated by the negative effect of the 70% ethanol on the middle lamella.

The presented results indicate that wood treated with a bio-friendly preparation based on the propolis extract and silicon compounds could be used in various application and also in variable humidity conditions.

## Figures and Tables

**Table 1:** Mean values of moisture content, density of wood before and after treatment, and retention of treated wood.

Wood Sample	Moisture Content (%)	Density of Wood before Treatment (kg/m^3^)	Density of Wood after Treatment (kg/m^3^)	Retention (kg/m^3^)
Control	10.2 ± 0.1	637.1 ± 17.2	637.1 ± 17.2	-
70% EtOH	9.8 ± 0.3	637.9 ± 17.9	626.0 ± 17.6	6.2 ± 0.2
EEP	8.0 ± 0.2	638.5 ± 17.6	699.0 ± 18.0	95.2 ± 3.1
MPTMOS/TEOS	8.2 ± 0.4	639.7 ± 19.1	650.0 ± 18.1	61.6 ± 2.6
EEP–MPTMOS/TEOS	8.1 ± 0.3	640.5 ± 16.1	720.0 ± 15.9	151.4 ± 6.6

Expressed as average ± standard deviations.

**Table 2:** Mean values of modulus of rupture (MOR), modulus of elasticity (MOE), and work to maximum load (WML) for treated wood in different moisture content (MC).

MC	Wood Sample	MOR (MPa)	MOE_L_ (MPa)	WML (J)
Dry	Control	124.0 ± 6.8 ^a^	16,876 ± 725 ^b^	0.806 ± 0.071 ^a^
	70% EtOH	126.6 ± 7.5 ^a^	16,537 ± 876 ^a,b^	0.847 ± 0.089 ^a,b^
	EEP	137.6 ± 6.8 ^b^	16,109 ± 677 ^a^	0.939 ± 0.067 ^b^
	MPTMOS/TEOS	124.4 ± 5.3 ^a^	15,724 ± 651 ^a,b^	0.792 ± 0.073 ^a^
	EEP–MPTMOS/TEOS	136.6 ± 7.1 ^b^	15,628 ± 844 ^a^	0.932 ± 0.066 ^b^
>FSP	Control	58.6 ± 4.3 ^a^	11,110 ± 666 ^a^	0.664 ± 0.129 ^a^
	70% EtOH	52.1 ± 4.2 ^a^	10,867 ± 837 ^a^	0.613 ± 0.074 ^a^
	EEP	72.3 ± 10.8 ^b^	11,720 ± 488 ^a^	0.761 ± 0.126 ^a^
	MPTMOS/TEOS	55.7 ± 3.7 ^a^	11,428 ± 406 ^a^	0.689 ± 0.123 ^a^
	EEP–MPTMOS/TEOS	102.7 ± 5.3 ^c^	13,100 ± 628 ^b^	1.042 ± 0.119 ^b^

Expressed as average ± standard deviations; ^a,b,c^ different superscripts denote a statistically significant (*p* < 0.05) difference between mean values according to Tukey’s HSD test.

## Data Availability

The data presented in this study are available on request from the corresponding author.
